# Clinical Applications of Corneal Cells Derived from Induced Pluripotent Stem Cells

**DOI:** 10.3390/biom15081139

**Published:** 2025-08-07

**Authors:** Yixin Luan, Aytan Musayeva, Jina Kim, Debbie Le Blon, Bert van den Bogerd, Mor M. Dickman, Vanessa L. S. LaPointe, Sorcha Ni Dhubhghaill, Silke Oellerich

**Affiliations:** 1 Ophthalmology Research Group, Faculty of Medicine and Pharmacy, Vrije Universiteit Brussel (VUB), 1090 Brussels, Belgium; luan.yixin@vub.be (Y.L.);; 2 Department of Ophthalmology, Universitair Ziekenhuis Brussel (UZB), 1090 Jette, Belgium; 3 Harvey & Bernice Jones Eye Institute, University of Arkansas for Medical Sciences, Little Rock, AR 72205, USA; 4 Antwerp Research Group for Ocular Science (ARGOS), Faculty of Medicine and Health Sciences, University of Antwerp, 2610 Wilrijk, Belgium; 5 Department of Ophthalmology, University of Utrecht, 3584 CM Utrecht, The Netherlands; 6 Department of Cell Biology–Inspired Tissue Engineering, MERLN Institute for Technology-Inspired Regenerative Medicine, 6229 ER Maastricht, The Netherlands

**Keywords:** vision impairment, corneal blindness, endothelial dystrophy, regenerative medicine, cell therapy, donor tissue shortage, corneal endothelial cells, corneal epithelial cells, stromal keratocytes

## Abstract

Corneal diseases are among the leading causes of blindness worldwide and the standard treatment is the transplantation of corneal donor tissue. Treatment for cornea-related visual impairment and blindness is, however, often constrained by the global shortage of suitable donor grafts. To alleviate the shortage of corneal donor tissue, new treatment options have been explored in the last decade. The discovery of induced pluripotent stem cells (iPSCs), which has revolutionized regenerative medicine, offers immense potential for corneal repair and regeneration. Using iPSCs can provide a renewable source for generating various corneal cell types, including corneal epithelial cells, stromal keratocytes, and corneal endothelial cells. To document the recent progress towards the clinical application of iPSC-derived corneal cells, this review summarizes the latest advancements in iPSC-derived corneal cell therapies, ranging from differentiation protocols and preclinical studies to the first clinical trials, and discusses the challenges for successful translation to the clinic.

## 1. Introduction

**Figure 1:**
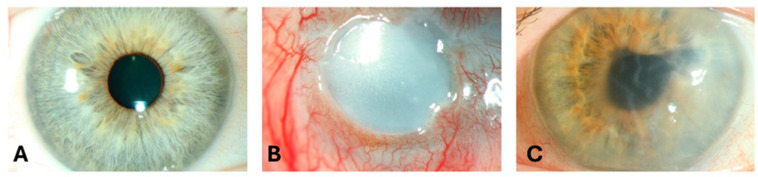
Slit-lamp images of (**A**) a 52-year-old female with a clear cornea, (**B**) an eye of a 52-year-old male patient with corneal blindness due to limbal stem cell deficiency, and (**C**) an eye of a 73-year-old male patient with severe visual impairment due to pseudophakic bullous keratopathy.

Corneal diseases are among the most common causes of reversible vision loss worldwide [1,2], second only to cataract (Figure 1) [3]. The causes of severe corneal damage are diverse and include dystrophies, infections, inflammatory diseases, and trauma, which can lead to irreversible corneal opacification and loss of vision [4,5]. The most effective method of improving vision in opaque corneas is through a corneal transplantation, which is called keratoplasty [6,7,8]. Keratoplasty, however, has some limitations. Firstly, it depends on the availability of donor corneas, which in turn requires an established eye banking infrastructure. Despite improvements in eye banking, a global shortage of corneal donor tissue persists, and this poses a significant challenge [1]. Secondly, as an allogenic procedure, corneal transplantation carries the risk of immune rejection, necessitating prolonged immunosuppressive therapy, typically in the form of topical corticosteroids, which can lead to secondary complications [9,10]. Moreover, in the more severe corneal conditions, such as total limbal stem cell deficiency (LSCD), transplantation alone is usually not effective, as the loss of limbal epithelial stem cells results in chronic epithelial defects and conjunctivalization, further compromising corneal integrity [11]. These challenges underscore the need for new therapeutic strategies to restore corneal anatomy and vision.

The use of corneal cells derived from induced pluripotent stem cells (iPSCs) offers great potential in addressing these challenges. Many limitations related to cell transplantation, which previously hindered clinical development, may be mitigated by employing iPSC-derived cells. Although the clinical application of iPSC-derived cells is a complex process [12,13], some cell replacement methods have already found their way to patients [14,15]. In this report, we will review the recent developments and challenges of using iPSC-derived corneal cells in corneal regenerative medicine.

## 2. Differentiation of iPSCs into Corneal Cells

**Figure 2:**
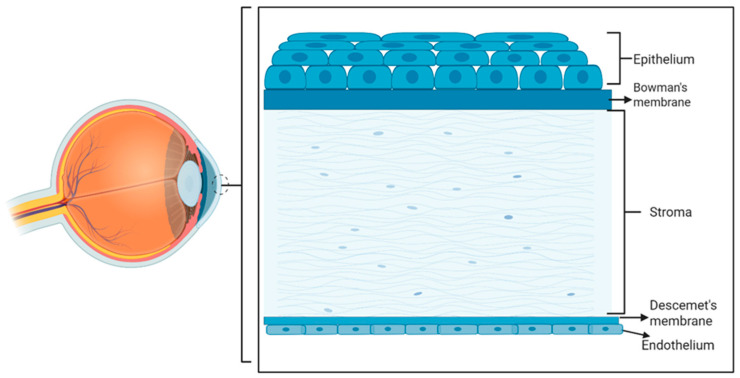
Schematic representation of the eye (left) and a zoom-in into the cornea with the epithelial cell layer on top (anterior corneal surface) and the endothelial cell layer at the bottom (posterior corneal layer). (Created in Biorender. LUAN, Y. (2025) https://BioRender.com/wwqqnpg, accessed on 2 July 2025).

The cornea is a transparent and avascular structure that provides the eye with 75% of its refractive power [16]. It consists of three cellular layers, the epithelium, stroma, and endothelium, which are separated by two basement membranes: Bowman’s membrane, located between the epithelial and stromal layers, and Descemet membrane, located between the stromal and endothelial layers (Figure 2).

During embryogenesis, the cornea arises from a combination of surface ectoderm and neural crest-derived mesenchymal stem cells [17]. The epithelial layer is derived from the ocular surface ectoderm [18,19], which gradually differentiates into corneal epithelial cells (CECs) [20,21]. Postnatally, the epithelium develops into two layers of basal epithelial cells and multiple layers of non-keratinized squamous epithelial cells [22,23,24]. The stromal and endothelial layers emerge from the migration of the ocular mesenchyme, which develops from neural crest cells (NCCs) [24]. Since the epithelium is derived from surface ectoderm, it retains highly proliferative properties, while the deeper stromal and endothelial cells, derived from NCCs, display a low proliferative potential and tend to remain quiescent in life.

Since their discovery in 2006, iPSCs have attracted broad research and clinical interest across medicine including ophthalmology, since these cells possess similar differentiation potential as embryonic stem cells (ESCs) but avoid the ethical concerns associated with ESC-based therapy [25,26]. In addition, patient-specific iPSCs can be generated from autologous somatic cells, reducing the risk of immune rejection [27]. However, given the complexity, cost, and time required for autologous therapies, allogeneic iPSC approaches are currently preferred for clinical applications. Alternatively, HLA haplobanks can provide iPSC lines with common haplotypes to match a wide range of patients, further minimizing immune incompatibility [28].

In the following sections, we will discuss the differentiation strategies for generating functional iPSC-derived CECs, stromal keratocytes, and corneal endothelial cells, together with their in vitro and in vivo functional validation.

### 2.1. Corneal Epithelial Cells

The epithelium is the outermost barrier of the cornea and consists of a variety of epithelial cell layers [29]. One of the purposes of the corneal epithelium is to protect the underlying stroma from the friction of the eyelids. With every blink, cells are shed, but in a healthy cornea, this loss is constantly replenished by proliferation from a population of stem cells located at the corneal limbus. These cells are known as corneal limbal epithelial stem cells [30]. If this population of stem cells is lost, the corneal epithelium can no longer be replenished, resulting in LSCD, which causes non-healing epithelial defects, leading to corneal haze, fibrosis, infections, perforations, and ultimately blindness [31].

LSCD can be treated by transplanting new corneal epithelial stem cells onto the damaged cornea [11]. This method, however, typically relies on using either allogenic donor corneal tissue or, in the case of unilateral disease, a portion of the limbus of the fellow eye. Using autologous tissue has higher success rates than allogeneic, but the prospect of risking the good fellow eye by removing a portion of the limbus can be daunting to the patient. Smaller biopsies can be used directly or cultured to generate a cultivated autologous stem cell transplantation [11]. Culturing autologous stem cells in vitro can present with technical challenges; however, therapeutic failure may also arise from factors such as graft integration issues, host immune response, or surgical complications, and can result in postoperative peripheral corneal neovascularization. The treatment can be repeated, but harvesting a biopsy more than twice confers a significant risk to the fellow eye [32]. The development of iPSC-derived limbal stem cells could offer considerable added value here as a new therapy that allows for large-scale cell production and repeated treatment [33].

Various differentiation protocols have been developed to guide iPSCs into corneal epithelial cells (CECs) [15,34,35,36,37], and will be discussed, along with the first preclinical studies and emerging clinical trials which have reported promising safety, efficacy, and translational potential of these cells for therapeutic applications [14,15].

#### 2.1.1. Protocols for Differentiation into Corneal Epithelial Cells

A number of protocols involving a series of defined steps that mimic ocular development have been developed to differentiate iPSCs into CECs [34,35]. Studies used different factors to promote the epithelial phenotype, such as conditioned medium, PA6 feeder cells, Bowman’s membrane, and amniotic membrane [38,39,40,41]. More recently, the differentiation of iPSCs into corneal epithelial cells has been advanced through the development of feeder-free protocols, defined cytokine supplementation, and biomaterial-supported culture systems [14,39,40,41]. Most protocols relied on the fact that the corneal epithelium develops from the ocular surface ectoderm [42]. Small molecule inhibitors (SB-505124 and IWP-2), blocking Wnt/β-catenin signaling pathways [43], in combination with fibroblast growth factor (FGF) [37], or collagen IV together with keratinocyte culture medium have been used with demonstrated positive effects [44]. Keratinocyte growth factor, epidermal growth factor (EGF), and insulin-like growth factor 1 have also shown potential to enhance epithelial commitment and promote stratification [45].

In culture, the choice of substrate is essential and a wide range of substrates including extracellular matrix components and synthetic materials have been tested and, among others, collagen IV appears to be a suitable substrate for CEC differentiation from iPSCs [46]. Hayashi et al. used a self-formed ectodermal autonomous multi-zone (SEAM) for the generation of ocular cells from iPSCs [47]. The SEAM culture method allows iPSCs to form concentric epithelial zones through self-organization, in contrast to the traditional protocols through the gradual addition of growth factors. The self-organization is achieved by cell–cell interactions and spatial arrangement under specific culture conditions. In their study, they used a 2D culture system with laminin LN511-E8 as a matrix for ocular cell growth with a serum-free differentiation medium to promote differentiation [47,48].

Various key cell markers have been suggested for CEC testing (Table 1). Obtaining the required purity of the differentiated CECs is a persistent challenge, as non-corneal cells often contaminate the culture. To address this, CD200-negative selection has been identified as an effective method for isolating pure corneal epithelial progenitors, and reducing the presence of unwanted retinal and neural cell populations [49]. In addition to this approach, several alternative strategies have also been applied to enrich iPSC-derived CECs, including marker-based sorting [49], functional clonal assays [50], genetic reporter systems [51], and matrix-guided selection methods [52]. A summary of these approaches is provided in Table 2.

#### 2.1.2. Preclinical Studies

Animal models have been used to evaluate the survival, integration, and functionality of iPSC-derived CECs (Table 3). For example, in rabbit models, transplanted iPSC-derived corneal epithelial sheets, which are sheet-like constructs composed of differentiated CECs, adhered successfully to the corneal surface, maintained transparency, and expressed key corneal epithelial markers such a CK3 and CK12, effectively mimicking native corneal epithelium [47,67]. Histological analyses confirmed that the transplanted cells formed a stratified epithelium resembling the native corneal structure [47]. Furthermore, in non-human primates, the long-term survival and functional integration of major histocompatibility complex-unmatched corneal epithelial cell sheets was demonstrated, indicating low immunogenicity and minimal immune rejection [68]. These findings suggest that allogeneic iPSC-derived CECs could be viable for clinical applications with only mild immunosuppressive treatment, which has major clinical advantage.

A significant concern in stem cell–based therapies is the potential risk of teratoma formation due to residual undifferentiated iPSCs. To mitigate this risk, researchers have employed fluorescence-activated cell sorting and antibody-based purification techniques to eliminate undifferentiated cells before transplantation (Table 2) [49]. Long-term tumorigenicity studies in immunodeficient mice have confirmed that purified iPSC-derived CECs did not form tumors, reinforcing their safety profile [14]. Additionally, the SEAM differentiation method has been shown to generate highly purified corneal epithelial progenitor cells, reducing the risk of tumorigenicity, as demonstrated in a recent clinical study [14]. These preclinical findings have laid a solid foundation for advancing iPSC-derived CEC therapies toward clinical applications.

#### 2.1.3. Emerging Clinical Trials

In 2019, the world’s first clinical trial using iPSC-derived CECs was launched in Japan (Table 3) [14]. In this first-in-human, single-arm study, iPSC-derived human leukocyte antigen-mismatched CEC sheets were transplanted in four patients with bilateral LSCD caused by various etiologies, including idiopathic LSCD, ocular mucous membrane pemphigoid, and toxic epidermal necrolysis. By design, the first two patients received systemic low-dose cyclosporin, while the subsequent two were managed only with topical steroids. This approach allowed investigators to assess the necessity of immune suppression for such allogeneic iPSC-derived grafts. Over a two-year follow-up period, all patients exhibited significant visual improvement without severe immune rejection or adverse effects. The first two patients, however, achieved better results than the subsequent two, suggesting that in the latter patients, subclinical chronic immunological rejection may have compromised the transplantation outcomes. Nevertheless, this study is a major step forward in regenerative medicine for corneal diseases, showing that iPSC-derived CECs could become a valuable treatment option, especially for patients with bilateral LSCD.

### 2.2. Corneal Stromal Keratocytes

The corneal stromal layer accounts for approximately 90% of the corneal thickness and plays a key role in maintaining its mechanical strength and transparency (Figure 2). It is primarily composed of highly organized collagen fibers, which are organized into lamellae that lie parallel to the surface of the cornea [69]. Corneal stromal keratocytes (CSKs) are the predominant cell type within the corneal stroma [70], and are responsible for the synthesis and organization of extracellular matrix components, including collagen types I, V, VI, and XII, as well as proteoglycans such as keratocan and lumican. These two proteoglycans, which both contain keratan sulfate, are highly expressed in CSKs and help maintain corneal transparency by keeping the collagen fibers well-organized and reducing light scattering [71,72].

As CSK density decreases, the production of these essential extracellular matrix components is compromised, leading to stromal disorganization and loss of transparency [73]. In eyes with keratoconus, a disorder that leads to progressive thinning and protrusion of the cornea, the number of CSKs decreases due to apoptosis [74], and in the advanced stages of keratoconus, severe stromal thinning, ectasia, and scarring resulting in severe visual impairment occur [75]. Corneal scarring is associated with activated CSKs responding to the diseased environment and transforming into myofibroblasts that deposit opaque fibrotic tissue [76]. In such cases, the only way to restore corneal clarity and function is currently through corneal transplantation [73]. However, the potential of iPSC-derived CSKs for stromal regeneration is also being evaluated as an alternative treatment for stromal opacifications [77].

#### 2.2.1. Protocols for Differentiation into Corneal Stromal Keratocytes

Keratocytes are known to proliferate in vitro in serum-supplemented medium [78,79]. Serum in culture medium, however, can lead to fibroblast differentiation and the downregulation of keratan sulfate proteoglycan expression, which is a unique property of CSKs [70,80,81]. By using iPSCs, the fibroblast differentiation may be avoided. Naylor et al. used a two-step protocol to differentiate iPSCs to CSKs [82]. First, they differentiated the iPSCs into intermediate NCCs, and then the cells were further differentiated from NCCs to CSK using two approaches: a three-dimensional (3D) culture system and a decellularized corneal tissue culture approach. The latter method resulted in better CSK morphology and extracellular matrix expression. The NCC-derived CSKs secreted keratan sulfate and keratocan, characteristic of CSKs. In contrast, 3D aggregates formed in suspension cultures of iPSCs, known as embryoid bodies, represent an alternative method to initiate differentiation into various lineages [83]. Joseph et al. described culturing iPSCs derived from embryoid bodies in keratocyte differentiation medium with DMEM/F12, FGF2, insulin, transferrin, and selenite to obtain keratocan (corneal keratocyte marker)-positive CSKs [84]. The role of the growth factor FGF2 in keratan sulfate proteoglycan production had been shown in bovine corneal cultures [85]. Similarly, in 2024 Chen et al. employed an embryoid body-based strategy to differentiate iPSCs into CSKs using keratocyte differentiation medium containing FGF2, which is essential for promoting keratocyte differentiation. The resulting cells expressed key keratocyte markers including ALDH1A1, lumican, and keratocan, and importantly lacked the expression of fibroblast markers such as ACTA2, suggesting a purer keratocyte phenotype [77].

Both the NCC-based and embryoid body-based methods can be used to derive CSK-like cells from iPSCs. The NCC can also be generated via embryoid bodies, and the NCC pathway more closely mimics natural development. The EB method is often considered simpler and involves fewer steps.

In addition, Foster et al. successfully developed a stepwise differentiation method to guide iPSCs into anterior ocular lineages, leading to the formation of 3D, multilayered corneal organoids that mimicked early corneal development [86,87]. These organoids contained epithelial, stromal, and endothelial-like layers, and could serve as a useful preclinical model for studying CSK development. However, modeling the stromal component remains challenging, and future translation may require a physical scaffold such as a decellularized cornea or synthetic carrier [88].

#### 2.2.2. Preclinical Studies and Emerging Clinical Trials

For iPSC-derived CSKs, as of May 2025, no preclinical studies or clinical trials have been reported. However, other stem cell-based strategies targeting corneal stromal regeneration have begun to show promise in a clinical settings. Alió et al. conducted the first human clinical trial exploring autologous adipose-derived mesenchymal stem cells (ADSCs) for corneal stromal regeneration in advanced keratoconus patients. In the initial phase I trial, ADSCs were injected into laser-created stromal pockets, resulting in good integration, no immune response, and increased stromal transparency and collagen deposition [89,90]. In a follow-up clinical study, Alió et al. evaluated three stromal regeneration strategies in nine patients with advanced keratoconus: (1) intrastromal injection of autologous ADSCs, (2) implantation of decellularized stromal lamina, and (3) implantation of stromal lamina re-cellularized with ADSCs. After one year, the re-cellularized group showed the greatest improvements in anterior stromal cell density and refractive outcomes, suggesting enhanced regenerative efficacy [91,92]. These outcomes point to the clinical potential of a similar approach using iPSC-derived CSKs in the future.

### 2.3. Corneal Endothelial Cells

Human corneal endothelial cells (CEnCs) form a monolayer of hexagonal cells [93], which have only limited proliferative ability in vivo [94]. The loss or damage of these cells compromises their essential pump function and can be detrimental to corneal transparency [93,95]. In healthy adults, CEnC density averages around 3000 cells/mm^2^ and gradually declines with age, disease, or surgical trauma [96]. When the endothelial cell density falls below a functional threshold, it leads to corneal edema and vision loss [97,98]. Currently, endothelial keratoplasty techniques such as Descemet stripping automated endothelial keratoplasty and Descemet membrane endothelial keratoplasty are the standard treatment for corneal endothelial dysfunction [7,8,99]. However, similar to other corneal surgical approaches, the global shortage of donor corneas eligible for transplantation remains a major limitation in clinical practice [1].

To address this, alternative techniques based on cell replacement and regenerative therapy are being explored. The state of the art has advanced to the point that in vitro expanded primary CEnCs have been translated into clinical applications. Recently, endothelial cell injection therapy has received market authorization in Japan [100], though it is important to note that the product still requires corneal donors. In that sense, pluripotent stem cells with an ability to generate corneal endothelial-like cells in vitro could provide an attractive non-donor dependent alternative. While early studies relied on ESCs [101], the emergence of iPSCs offers a more ethically acceptable and clinically feasible alternative [102,103,104].

#### 2.3.1. Protocols for Differentiation into Corneal Endothelial Cells

Currently, there are several studies focusing on generating CEnCs from iPSCs for cell replacement therapy [105,106,107]. The main challenges for developing protocols for CEnC differentiation from iPSCs are our limited understanding of the human CEnC development process and the signals regulating their proliferation and maturation [108]. This is compounded by difficulties in defining biomarkers for human CEnCs [109]. Due to the lack of clear, unambigous CEnC biomarkers and a different level of marker expression compared to primary CEnCs, the iPSC-derived CEnCs are often referred to ‘endothelial-like’ cells.

Similarly to epithelial and stromal culture, two-stage differentiation protocols for CEnCs have also been described. In the first step, iPSCs are guided into NCCs, mainly by the influence of BMP4, FGF2, and ROCK inhibitors, and then further differentiated into endothelial-like cells [110]. Chambers et al. established a highly efficient neural induction protocol using dual-SMAD inhibition (SB431542 and LDN193189), which has since been widely applied to generate NCCs from iPSCs for various lineages, including ocular cells [106]. These NCCs express characteristic markers such as SOX10, FOXD3, and PAX3, and can be further differentiated into endothelial-like cells under defined conditions [111].

Zhao et al. reported a multi-step approach for the differentiation of iPSCs and ESCs to CEnCs, which took the key molecular signals, such as Wnt/β-catenin activation, TGF-β inhibition, and ROCK pathway modulation, into consideration [112]. In contrast to conventional multi-step protocols involving NCC intermediates, Hatou et al. developed a simplified protocol to generate corneal endothelial-like cells directly from iPSCs, bypassing the neural crest stage [113]. Their method relied on a xeno-free, chemically defined medium containing key supplements including basic FGF, EGF, insulin–transferrin–selenium-A, ascorbic acid, and the ROCK inhibitor Y-27632. Under these conditions, the iPSCs were successfully differentiated into endothelial-like cells within 28 days. The cells tolerated cryopreservation and thawing well, maintaining their identity and morphology. Importantly, the proportion of undifferentiated iPSCs was kept below 0.01%, suggesting a strong safety profile for translational use. Additionally, Grönroos et al. developed a xeno-free, stepwise protocol to generate corneal endothelial-like cells using small molecules (SB431542, CHIR99021, and retinoic acid) under chemically defined conditions, further expanding clinically relevant approaches [114].

Chen et al. applied a different strategy. They developed a stepwise differentiation method involving neural crest induction followed by small-molecule screening to enhance CEnC marker expression [104]. Maurizi et al. further showed that a small molecule used in iPSC-differentiation (GSK-3β inhibitor) could maintain the functional phenotype and hexagonal morphology of native CECs [115]. Functional assays have shown that iPSC-derived endothelial-like cells exhibit hexagonal morphology, maintain pump activity, and form tight junctions, resembling native CEnCs [105].

Recently, Grönroos et al. further applied their protocol for 3D bioprinting of iPSC-derived endothelial-like cells using hyaluronic acid-based bioinks, supporting future clinical applications [116].

#### 2.3.2. Preclinical Studies

The therapeutic potential of iPSC-derived CEnC has been evaluated in various animal models, including rats, rabbits, and monkeys (Table 3) [107,113].

Among these models, Hatou et al. tested the efficacy and safety of iPSC-derived corneal endothelial-like cells in a non-human primate model of corneal edema [113]. Monkeys received 8 × 10^5^ cryopreserved cells by anterior chamber injection, together with a ROCK inhibitor to support cell adhesion. Within two weeks, the treated eyes showed recovery of corneal clarity and formation of a hexagonal monolayer. Immunostaining with a human-specific marker confirmed cell engraftment, and gene expression analysis showed similarity to native human CEnCs.

To further assess functional outcomes under surgical conditions, several studies have used rabbit models, which allow for intraocular procedures similar to standard endothelial keratoplasty surgery and short-term evaluation [117]. Sun et al. transplanted iPSC-derived CEnCs into a lapine model with a 7 mm diameter endothelial defect [103]. The treated eyes showed reduced corneal edema and significantly thinner central corneal thickness compared to the control group, suggesting functional recovery. Hsueh et al. also tested the therapeutic potential of iPSC-derived human corneal endothelial progenitor-like cells in a lapine model of corneal endothelial dysfunction induced by benzalkonium chloride [102]. A suspension of 1 × 10^6^ iPSC-derived human corneal endothelial progenitor-like cells were injected into the anterior chamber and led to a clear improvement in corneal transparency and pachymetry within 2 to 4 weeks. The cornea remained transparent for more than 210 days, and subsequent histological staining showed a monolayer of cells that expressed the typical corneal endothelial markers. Tumorigenicity tests in severe combined immunodeficient mice showed no evidence of tumor formation. These results indicate that iPSC-derived human corneal endothelial progenitor-like cells can repair corneal endothelial damage and may be safe for long-term use. In contrast to the suspension injection approach, Chi et al. applied the iPSC-derived endothelial-like cells to a thermosensitive hydrogel scaffold and implanted them in a rabbit model [118]. The treated eyes showed improved corneal transparency, cell retention, and monolayer formation compared to cell injection alone. No signs of inflammation or structural damage were observed, suggesting reasonable biocompatibility and functional recovery.

Two preclinical studies have also been performed in rats [15]. In the first tumorigenicity study, a high dose of 1 × 10^6^ endothelial-like cells was injected into the anterior chamber and was observed for 52 weeks. Histological analysis showed no tumor formation, and the proliferation marker Ki67 was sparsely expressed. In the second study, a lower dose of 2.5 × 10^4^ cells was used and PCR analysis showed no trace of transplanted cells in major organs. Histological and OCT4 staining confirmed the absence of undifferentiated cells. These results demonstrated the longer-term safety of these cells and directly supported the initiation of a first-in-human clinical trial.

#### 2.3.3. Emerging Clinical Trials

In the first-in-human study, corneal endothelial-like cells were derived from a clinical-grade iPSC line (QHJI01s04) provided by the Center for iPS Cell Research and Application (CiRA), Kyoto University [15]. The cells were cultured in xeno-free conditions using Stemfit AK03N medium on iMatrix-511-coated plates, and differentiation was induced with a defined corneal endothelial induction medium. The corneal endothelial-like cells exhibited typical endothelial features, with a strong expression of Na^+^/K^+^-ATPase, ZO-1, N-cadherin, and PITX2. Flow cytometry confirmed that over 97% of cells expressed key markers, indicating efficient and clinically compatible differentiation and a residual undifferentiated iPSC rate of less than 0.01% in their clinical-grade iPSC-derived corneal endothelial cells was reported. Based on these results, the cells were applied in a clinical setting to evaluate their therapeutic potential. To assess clinical feasibility, Hirayama et al. injected 8 × 10^5^ cells into the anterior chamber of a patient with bullous keratopathy [15]. At the one-year follow-up, the patient showed improved corneal clarity and best-corrected visual acuity, with no serious adverse events or signs of tumor formation. Postoperative genomic analysis revealed a de novo heterozygous deletion in the *EP300* gene in the transplanted cells, but no clinical consequences were observed. This first-in-human study demonstrated the therapeutic potential and short-term safety of iPSC-derived CEnC therapy, while emphasizing the need for continued genomic monitoring in clinical applications.

**Table 3:** Overview of preclinical studies and clinical trials with iPSC-derived corneal cells. (CEC = corneal epithelial cells, CEnC = corneal endothelial cells, LSCD = limbal stem cell deficiency).

**Preclinical Studies**				
**Corneal Cell Type**	**Animal Model**	**Cell Delivery Method**	**Main Findings**	**Reference**
CEC	Non-human primates (LSCD model)	Transplantation of cell sheets	Long-term (1 year) post-transplantation survival in monkeys; no rejection	Yoshinaga et al., 2022 [68]
CEC	Nude mice	Subcutaneous transplantation of cell sheet	No tumor formation	Soma et al., 2024 [14]
CEC	Rabbit (LSCD model)	Transplantation of cell sheet	Restored barrier function	Hayashi et al., 2016 [47]
CEnC Precursors	Rabbit (Bullous keratopathy model)	Cell injection into anterior chamber	Monolayer formation of hexagonal cells; reduced corneal edema	Li et al., 2022 [119]
CEnC	Non-human primates (Bullous keratopathy model)	Cell injection into anterior chamber	Monolayer formation of hexagonal cells; reduced corneal edema	Hatou et al., 2021 [113]
CEnC	Rabbit (Bullous keratopathy model)	Cell injection into anterior chamber	Monolayer formation of hexagonal cells; reduced corneal edema	Hsueh et al., 2025 [102]
CEnC	Rabbit (Bullous keratopathy model)	Cell injection into anterior chamber	Monolayer formation of hexagonal cells; reduced corneal edema	Sun et al., 2021 [103]
CEnC	Rabbit (Bullous keratopathy model)	Transplantation of cells on a thermosensitive hydrogel scaffold	Facilitated restoration of corneal clarity and function in a rabbit model of corneal endothelial dysfunction	Chi et al., 2025 [118]
CEnC	Rats	Cell injection into anterior chamber	No tumor formation	Hirayama et al., 2025 [15]
**Clinical Trials**				
**Corneal Cell Type**	**Surgeon Indication**	**Cell Delivery Method**	**Main Finding**	**Reference**
CEC	LSCD	Transplantation of iPSC-derived epithelial cell sheets onto corneal surface	iPSC-derived epithelial sheets survived for up to 1 year; no serious adverse events related to the grafts	Soma et al., 2024 [14]
CEnC	Bullous keratopathy	Cell injection into anterior chamber	Corneal transparency improved and central thickness decreased; visual acuity improved in 2 of 3 patients	Hirayama et al., 2025 [15]

## 3. Challenges for Clinical Applications of iPSC-Derived Corneal Cells

While first preclinical and clinical studies show promising results for potential clinical applications of iPSC-derived corneal cells, a number of challenges must be overcome for a successful translation into clinical practice. These range from continuous safety and regulatory concerns to scalability and cost-effectiveness.

### 3.1. Teratogenicity and Safety

Safety concerns related to the clinical application of iPSC-derived corneal cells include the production of fully differentiated, pure, clinical grade cells, and the risk of tumor formation [120]. The tumorigenic potential of iPSC-generated cells is related to their pluripotent nature and will require extensive evaluation of each iPSC-derived cell type and the iPSC line [121,122]. In the aforementioned first-in-human clinical trial [15], thorough genomic screening was conducted before the transplantation, yet an unexpected mutation was still discovered afterwards. The mutation involved a spontaneous deletion in the *EP300* gene, which had not been detected earlier. In a clinical trial with iPSC-derived retinal pigment epithelial cells for the treatment of age-related macular degeneration, single nucleotide mutations were also observed before the planned treatment [123]. These events highlight the importance of vigorous pre- and post-treatment safety monitoring as requested by regulatory agencies [124,125].

### 3.2. Immune Response and Compatibility

Immune compatibility remains another key challenge, especially when considering allogeneic therapies. Although iPSC-derived corneal cells typically express lower levels of HLA class I/II molecules than somatic cells, potentially reducing immune rejection risks [14,103], the concern about immune rejection cannot be entirely eliminated. Hatou et al. demonstrated that iPSC-derived endothelial-like cells restored corneal clarity in monkeys without signs of immune rejection during the observation period. However, the monkeys were immunosuppressed, and the follow-up was limited to six months, which may not fully exclude long-term rejection risks [113]. Similarly, Soma et al. reported that the transplantation of iPSC-derived corneal epithelial sheets did not cause rejection, even in HLA-mismatched settings. The authors reasoned that this was most likely due to a relatively low expression of HLA class I and II, and the lack of immunocompetent cells in the graft [14].

To avoid any immune-related problems, “patient-specific” autologous iPSC-derived corneal cells could be generated or an immune-evasive approach with allogeneic cells can be considered. While this has become increasingly feasible in recent years, particularly in countries like the US, the autologous iPSC-derived cells would need a significant amount of time to be generated and would not allow for cost-efficient bulk production [126]. Interestingly, it has been shown that complete HLA-matching is not a requirement and selecting a certain number of HLA-matched iPSC lines may be sufficient to be used for obtaining corneal cells [127,128,129]. While creating universal donor iPSC lines has the potential to be immune-matched to a great proportion of the population [128], generation of such HLA-matched iPSC lines still is more time-consuming and expensive compared to an allogenic approach. 

For endothelial keratoplasty HLA-matching is not common and it should also be kept in mind that with the introduction of the more selective lamellar corneal transplantation techniques, it was shown that the percentage of immune reactions after corneal transplantation, which is already low due to the immune privilege of the eye, dropped to around 3% after Descemet membrane endothelial keratoplasty [130]. Assuming that for the treatment of endothelial dysfunction similar rejection rates might occur with new corneal therapeutic options based on iPSC-derived corneal cells, an allogeneic iPSC approach may well be justified.

### 3.3. Cost-Effectiveness and Regulatory and Ethical Considerations

Safety and immune compatibility aside, scalability, manufacturing, financial, regulatory, and ethical aspects remain challenges in translating iPSC-derived corneal therapies into clinical practice. Producing clinical-grade iPSC-derived cells is a time-consuming, resource-intensive process that needs to adhere to strict GMP guidelines [15]. In combination with stringent testing requirements to ensure the safety of the cell therapy, these factors are resulting in a costly production process [131]. In order to contain the associated costs, many of which are influenced by regional regulatory requirements and the commercial viability of the company offering the therapies, allogeneic instead autologous cells may be used to enable ‘bulk’ production and reduce testing costs for successful translation to the clinic [110].

In order to meet regulatory requirements, all components for the production of the iPSC-derived corneal therapeutic product are strongly recommended to be xeno-free that is free of materials derived from non-human animal sources. Even though the required GMP-grade, commercial xeno-free options are generally available with adequate safety and traceability, this aspect can still pose a regulatory challenge.

The clinical use of iPSC-derived corneal cells involves strict regulatory review and ethical considerations. Like for corneal endothelial cell therapy based on primary endothelial cells, Japan is at the forefront for clinical trials for iPSC-derived corneal therapies and the first clinical trials for iPSC-derived corneal epithelial and endothelial cells were approved by local certified special committees and the Health Science Council of the Japanese Ministry of Health, Labor and Welfare. Countries, however, differ widely in their regulatory approaches to iPSC-based therapies, with Japan adopting a relatively flexible conditional approval system [14,15,110,124], the EU maintaining more conservative pathways, and the United States requiring robust evidence under FDA oversight [131]. As gene-edited or universal iPSC products continue to emerge, global regulatory harmonization and long-term monitoring frameworks will be increasingly important.

## 4. Future Directions

Preliminary clinical trials have demonstrated the feasibility of transplanting iPSC-derived corneal epithelial and endothelial cells with promising clinical outcomes [14,15]. The findings of these studies show the clinical potential of iPSC-derived corneal cells as donor tissue substitutes. Despite these advances, important scientific and technical challenges remain for widespread translation into the clinic. For epithelial and endothelial cells, efforts should focus on improving manufacturing efficiency and validating long-term functional stability in vivo [113]. In contrast, for corneal stromal cells the field is still in early development. This is because a true stromal replacement would require some bioengineering, since the cells are only a small fraction of the material needed. Certainly, 3D structures are needed and not monolayers as for the epithelium and endothelium. Current research focuses on differentiating iPSCs into keratocyte-like cells and reconstructing a transparent stromal matrix to restore corneal transparency.

## 5. Conclusions

Induced PSC-derived corneal cells offer a scalable and standardized cell source that has the potential to address global donor shortage. To realize the full clinical potential of iPSC-based corneal therapies, ongoing collaboration between researchers, clinicians, and regulators is necessary and will hopefully lead to a viable clinical alternative in the near future to alleviate the worldwide shortage of corneal donor tissue.

## Figures and Tables

**Table 1:** Overview of selected markers proposed for the identification of differentiated corneal epithelial cells.

Biomarker	Category	Function	Key References
CK3, CK12	Differentiation markers	Specific terminal differentiation marker of CECs. Limbal epithelial basal layer lacks the expression of these markers	Joe et al., 2014 [53] Mikhailova et al., 2014 [37] Hayashi et al., 2012 [41]
Connexin 43	Negative marker	Involved in cell–cell contact; not expressed during early CEC proliferation	Chen et al., 2006 [54] Li et al., 2014 [55]
CK4, CK13	Negative marker	Conjunctival epithelial markers; not expressed in mature corneal epithelium; markers of non-keratinized epithelium	Ramos et al., 2015 [56]
CK15	Progenitor marker	Expressed in limbal basal layer; marker of corneal progenitor status	Mikhailova et al., 2014 [37]
p63/ΔNp63α	Stem cell marker	Expressed in limbal basal cells; ΔNp63α is a key indicator of regenerative/stem-like capacity	Pellegrini et al., 2001 [57] Mikhailova et al., 2014 [37] Hanson et al., 2013 [40] Soma et al., 2024 [14] Li et al., 2024 [58] Vattulainen et al., 2021 [59]
PAX6	Lineage transcription factor	Master regulator of eye development; activates CK12 and CK3 expression during iPSC corneal lineage commitment; necessary for early development of eyes	Grindley et al., 1995 [60] Sasamoto et al., 2016 [61] Hayashi et al., 2012 [41] Cieślar-Pobuda et al., 2016 [34]
ABCB5	Limbal stem cell marker	A potential role in limbal epithelial stem cell quiescence and wound healing; ATP-binding transporter expressed in true limbal epithelial stem cells	Ksander et al., 2014 [62] Vattulainen et al., 2021 [59]
ABCG2	Stem/progenitor marker	Transiently expressed in early limbal progenitors	Li et al., 2014 [55] Cieślar-Pobuda et al., 2016 [34]
CK14	Basal epithelial marker	Expressed in basal progenitor layer of limbal and conjunctival epithelium; seen in early iPSC-CEC differentiation	Shalom-Feuerstein et al., 2013 [63] Zhang et al., 2017 [64] Cieślar-Pobuda et al., 2016 [34] Li et al., 2024 [58]
MUCIN-16	Surface mucin protein	Important for corneal epithelial barrier function	Soma et al., 2024 [14]
p75NTR (CD271)	Limbal stem cells marker	Downregulated upon differentiation; involved in NGF-mediated support of limbal stem cell phenotype	Kolli et al., 2019 [65] Yamamoto et al., 2010 [66]

**Table 2:** Overview of approaches to enhance the purity of iPSC-derived corneal epithelial cell populations.

Method Type	Specific Markers	Platform	Target Cell Type	Enrichment Logic	Key References
Surface Marker-Based Sorting	SSEA-4^+^/ITGB4^+^/TRA-1-60^−^	FACS	Enriched cell type	Positive selection for surface ectodermal markers; negative for undifferentiated iPSCs	Hayashi et al., 2018 [49]
Surface Marker-Based Sorting	CD200^−^	MACS/FACS	Enriched cell type	Removes undifferentiated iPSCs and non-CE cells expressing CD200	Hayashi et al., 2018 [49]
Functional Clonal Assay	ABCB5^+^	FACS + Holoclone assay	Corneal epithelial stem cells	ABCB5^+^ population forms holoclones; shows high ΔNp63 expression	Watanabe et al., 2021 [50]
Genetic Reporter System	p63-EGFP knock-in	Live imaging/FACS	p63^+^ epithelial progenitor cells	EGFP signal indicates active p63 promoter in stem/progenitor population	Kobayashi et al., 2017 [51]
Matrix Adhesion Selection	LN332E8 matrix	Selective adhesion	CECs	CECs prefer LN332E8 over LN211E8; allows selective attachment and expansion	Shibata et al., 2018 [52]

## Data Availability

Not applicable.

## Abbreviations

The following abbreviations are used in this manuscript:

3D	three-dimensional
ADSC	adipose-derived mesenchymal stem cell
CEC	corneal epithelial cell
CEnC	corneal endothelial cell
CSK	corneal stromal keratocyte
ESC	embryonic stem cell
EGF	epidermal growth factor
FGF	fibroblast growth factor
GMP	good manufacturing practice
HLA	human leukocyte antigen
iPSC	induced pluripotent stem cell
LSCD	limbal stem cell deficiency
NCC	neural crest cell
SEAM	self-formed ectodermal autonomous multi-zone
